# Immune shifts in inflammatory skin diseases

**DOI:** 10.1016/j.xjidi.2026.100499

**Published:** 2026-06-04

**Authors:** Hugo Cometto, Fanny Saidoune, Jeremy Di Domizio, Michel Gilliet

**Affiliations:** 1 Department of Dermatology, Lausanne University Hospital (CHUV), University of Lausanne, Lausanne, Switzerland

**Keywords:** Atopic dermatitis, Immune shifts, Psoriasis, Paradoxical reaction, Precision dermatology, Resistance

## Abstract

Inflammatory skin diseases are driven by dominant immune programs, including T helper (Th)1, Th2, Th17, type I IFN, and neutrophilic pathways, which shape responsiveness to targeted immunotherapies. Molecular profiling has revealed that these diseases frequently harbor additional, coexisting immune programs and that dominance among pathways can shift over time, either spontaneously or in response to therapy. Such immune shifts, which limit therapeutic effectiveness, often occur in the absence of clinically evident morphological changes. In this review, we describe the molecular immune landscape of inflammatory skin diseases, with an emphasis on psoriasis and atopic dermatitis, and illustrate the immune shifts that underlie therapy-resistant disease states and paradoxical reactions. We propose a model of an immune continuum governed by a dynamic equilibrium among inflammatory pathways that is susceptible to displacement. Finally, we highlight molecular immune profiling as a critical tool for detecting such shifts and enabling rational therapeutic adaptation.

## Molecular Immune Landscape of Inflammatory Skin Disease

Inflammatory skin diseases can be positioned within a molecular immune landscape defined by dominant cytokine programs (Seremet et al, 2024). Within this framework, psoriasis is distinguished by a strong T helper (Th)17–driven transcriptional signature that clearly separates it from other major inflammatory dermatoses. These include Th2-dominant atopic dermatitis (AD), type I IFN–driven disorders such as cutaneous lupus erythematosus, Th1-predominant diseases such as lichen planus, and eosinophilic and neutrophilic dermatoses characterized by macrophage- and neutrophil-associated gene expression (Seremet et al, 2024). Molecular profiling studies consistently demonstrate that the dominant immune pathway active in lesional skin is a key determinant of therapeutic responsiveness. Alignment between a therapeutic target and the prevailing immune pathway is required for clinical efficacy. Conversely, therapeutic mismatches, defined as targeting pathways that are not immunologically dominant, are associated with poor clinical responses (Di Domizio et al, 2025; Seremet et al, 2026; Seremet et al, 2024). Importantly, realigning treatment strategies with the underlying immune profile can restore therapeutic efficacy. Collectively, these findings establish the conceptual foundation for precision dermatology, in which molecular immune profiling informs rational and individualized treatment selection.

## Additional Immune Pathways in Disease Endotypes

### Psoriasis

Comprehensive molecular profiling across multiple clinical subtypes of psoriasis has revealed a shared dominant Th17 activation program accompanied by 2 additional inflammatory modules (Conrad and Gilliet 2018; Griffiths et al, 2021). One subset exhibits a combined Th17 profile and type I IFN signature, characterized by the upregulation of IFN-α/β cytokines and IFN-α/β-stimulated genes. A second subset is defined by Th17 activation, together with enhanced expression of neutrophil-associated genes. Collectively, these findings support the existence of 3 major molecular endotypes in psoriasis. The Th17-only endotype is pathogenetically linked to dermal dendritic cell activation and IL-23–mediated Th17 expansion with production of IL-17A/F (Figure 1). This endotype predominates in stable, chronic plaque psoriasis. The Th17-type I IFN endotype reflects the coexistence of Th17 and type I IFN signaling and is associated with activation of skin-infiltrating plasmacytoid dendritic cells (pDCs), which produce IFN-α/β, a pathway implicated in early disease initiation (Lande et al, 2007; Nestle et al, 2005). Clinically, this endotype characterizes acute and unstable disease states, including erythrodermic psoriasis and the inflammatory margins of newly emerging plaques (Conrad and Gilliet 2018). Notably, approximately half of chronic plaque psoriasis lesions also display detectable IFN signatures, suggesting episodic reactivation of type I IFN–driven inflammation even in established disease. Finally, the Th17 + neutrophilic autoinflammation (ie, TH17 + neutro) endotype combines a Th17 profile with variable degrees of neutrophilic inflammation, elevated IL-1 family cytokines (including IL-1α and IL-1β), IL-36 signaling, and marked neutrophil infiltration (Figure 1). This endotype is most frequently associated with pustular variants, including generalized pustular psoriasis and palmoplantar pustular psoriasis (Baldo et al, 2024).

**Figure 1:**
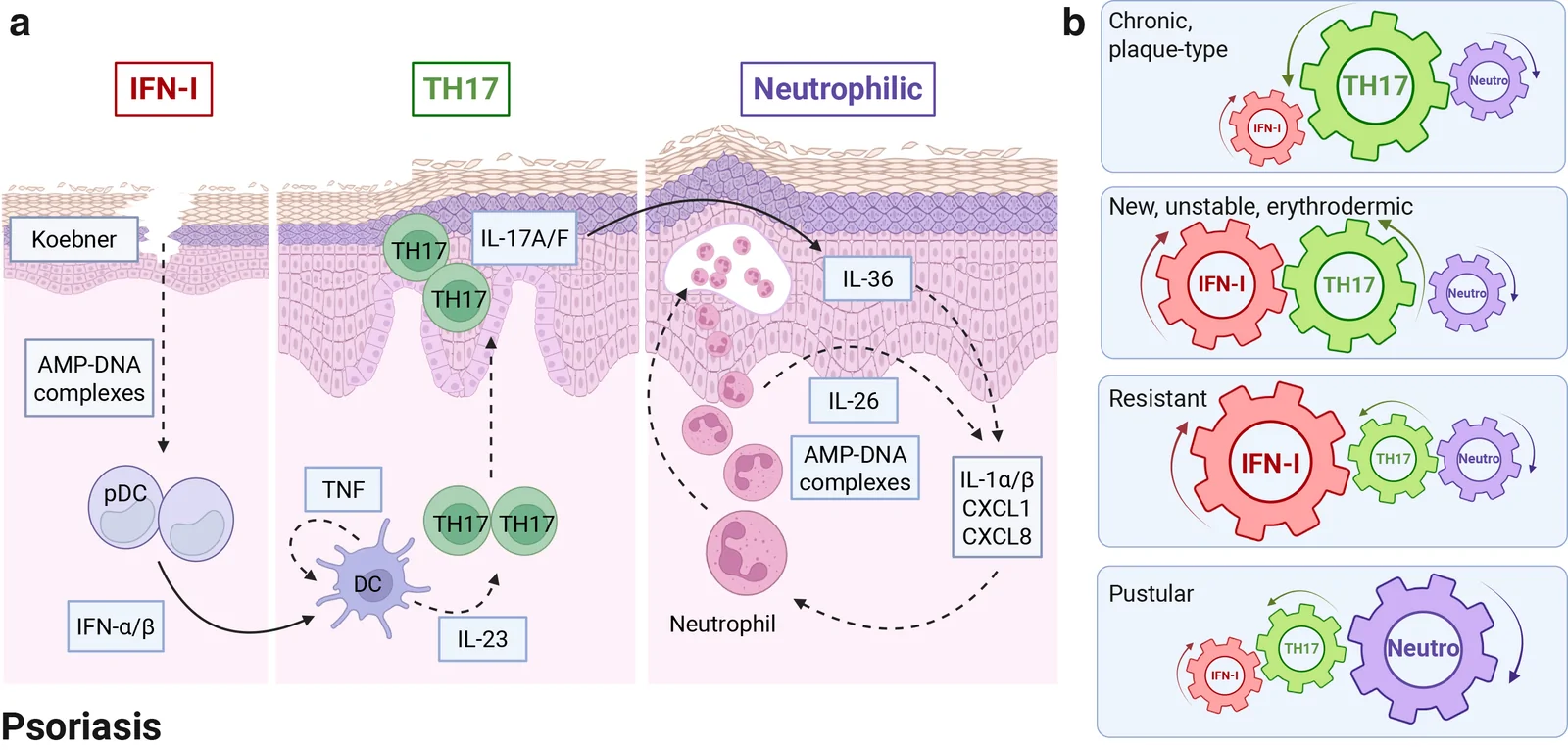
**Immune pathways underlying psoriasis pathogenesis and their association with clinical endotypes.** (**a)** Skin injury (Koebner phenomenon) induces the formation of AMP–DNA complexes that activate pDCs to produce type I IFN-α/β (left panel). This early innate immune activation promotes myeloid DC maturation and the release of TNF and IL-23, driving Th17 cell differentiation and the secretion of IL-17A/F. These cytokines act on keratinocytes to induce epidermal hyperplasia and sustain chronic plaque formation (middle panel). IL-17–activated keratinocytes produce IL-36 and IL-1, which stimulate the expression of neutrophil-attracting chemokines, including CXCL1 and CXCL8. Infiltrating neutrophils release IL-26, which directly activates keratinocytes and can form AMP–DNA complexes, thereby amplifying inflammatory signaling. This feed-forward loop enhances neutrophil recruitment and promotes pustular inflammation (right panel). **(b)** Clinical psoriasis endotypes are characterized by the relative dominance of these immune pathways. Chronic plaque-type psoriasis is predominantly Th17 driven, with minor contributions from type I IFN and neutrophilic pathways. New, unstable, or erythrodermic psoriasis exhibits heightened type I IFN activation alongside Th17 responses. Treatment-resistant psoriasis is associated with persistent type I IFN activity and mixed pathway engagement, whereas pustular psoriasis is defined by marked neutrophilic predominance with contributions from Th17 and type I IFN signaling. AMP, antimicrobial peptide; DC, dendritic cell; pDC, plasmacytoid dendritic cell; Th17, T helper 17.

Psoriasis susceptibility gene variants are enriched at loci involved in Th17 responses, including *IL17RA*, *IL23R*, *IL23A*, and *IL12B* (Dand et al, 2025; Tsoi et al, 2015); at *TYK2* and *RIGI* loci involved in type I IFN signaling (Dand et al, 2017; Tsoi et al, 2017); and at *IL36RN* and *MPO*, which participate in IL-36–driven and neutrophil-mediated inflammatory pathways (Marrakchi et al, 2011; Vergnano et al, 2020). Together, these findings support a genetic predisposition to dysregulated type I IFN, neutrophilic, and Th17 immune responses across different psoriasis endotypes.

### Atopic dermatitis

AD is a heterogeneous Th2-driven inflammatory disease characterized by a shared Th2 activation program (Figure 2) (Bieber 2008; Brunner et al, 2017). This canonical Th2 profile predominates in early-onset, extrinsic, IgE-high disease. Functionally, Th2 cytokines promote epidermal barrier dysfunction, IgE class switching, eosinophil recruitment, and pruritus. Beyond this Th2 profile, additional immune modules have been identified. A Th2 + Th17 endotype is observed in pediatric AD (Esaki et al, 2016) and in patients with AD who are of Asian origin (Noda et al, 2015), highlighting age- and ethnicity-dependent immune variation. The Th2 + Th17 endotype is also prominent in nummular forms of atopic eczema (Böhner et al, 2023). A Th2 + Th22 endotype, marked by elevated IL-22 expression, contributes to epidermal hyperplasia and lichenif ication, particularly observed in chronic adult disease (Gittler et al, 2012). Another contributor of disease chronicity is the Th1 module characterized by increased IFN-γ signaling (Thepen et al, 1996). Together, these immune endotypes provide a mechanistic explanation for the marked clinical heterogeneity of AD.

**Figure 2:**
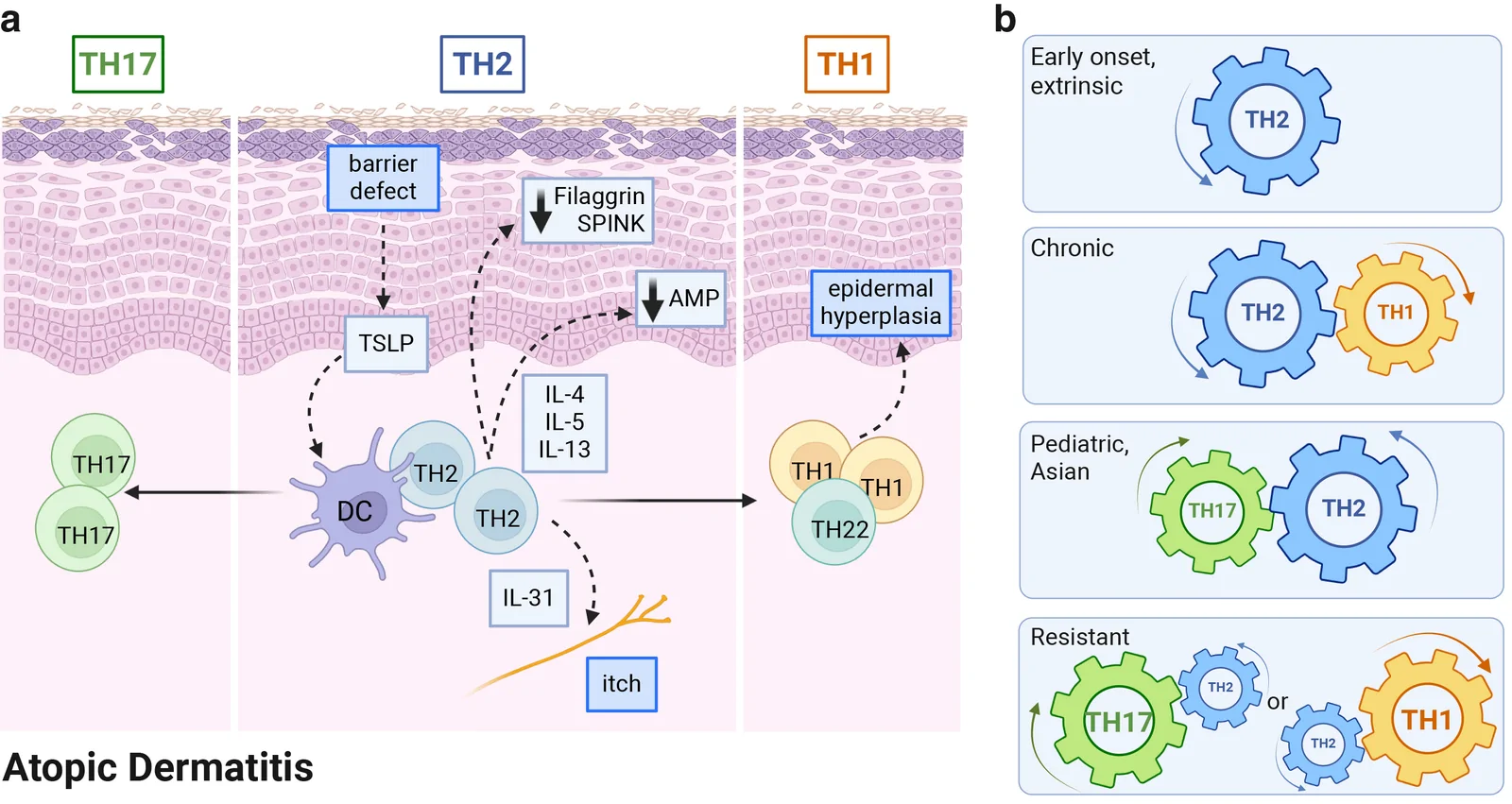
**Immune pathways underlying atopic dermatitis pathogenesis and their association with clinical endotypes.** (**a)** Skin barrier defects, including reduced FLG and SPINK expression, lead to impaired AMP production and enhanced transepidermal antigen penetration (left and middle panels). Barrier disruption promotes keratinocyte-derived TSLP release, which activates DCs and drives Th2 polarization. Th2 cells produce IL-4, IL-5, and IL-13, further suppressing barrier proteins and AMPs, thereby reinforcing barrier dysfunction in a feed-forward loop. Th2 cytokines also induce IL-31, contributing to pruritus. In addition to Th2 signaling, variable contributions from Th17 and Th22 pathways are observed. Th17 cells may be present, particularly in specific endotypes (left panel), whereas Th22 and Th1 responses contribute to epidermal hyperplasia and chronic inflammation (right panel). **(b)** Clinical endotypes of atopic dermatitis reflect the relative dominance of these immune pathways. Early onset, extrinsic atopic dermatitis is primarily Th2 driven. Chronic disease shows persistent Th2 activation with increasing Th1 involvement. Pediatric and Asian endotypes demonstrate combined Th2 and Th17 activation. Treatment-resistant disease may exhibit either enhanced Th17 skewing or a shift toward Th1 dominance alongside ongoing Th2 activity. AMP, antimicrobial peptide; DC, dendritic cell; Th, T helper; TSLP, thymic stromal lymphopoietin.

Variants that increase susceptibility to AD are highly enriched at the *FLG* locus and at loci associated with Th2 immune polarization and type 2 cytokine signaling, including *IL-4,* *IL-13, IL-4Rα, signal transducer and activator of transcription* *(STAT)-6, and thymic stromal lymphopoietin (TSLP)* genes (Ferreira et al, 2017; Hirota et al, 2012; Paternoster et al, 2015). Some of these loci also influence both Th2 and Th17 responses (Weidinger et al, 2013) as well as Th1 and Th22 responses (Sliz et al, 2022). Collectively, these data indicate a genetic predisposition toward enhanced type 2 immune activity and skin barrier dysfunction, with variable involvement of Th1, Th17, and Th22 responses across different AD endotypes.

### Other diseases

Immune endotypes of other inflammatory dermatoses are less well-characterized. Lupus erythematosus is defined by a core type I IFN endotype but appears to have additional contributions from Th1-driven inflammation (Wenzel et al, 2005), B-cell signatures, and tissue-remodeling programs in chronic disease (Zheng et al, 2022). Lichen planus is dominated by Th1 responses (Hwang et al, 2025) but may be modulated by variable type I IFN activity (Wenzel et al, 2006).

## Immune Shifts in Therapy-Resistant Disease

Whether these additional immune pathways interfere with responsiveness to biologics targeting canonical disease pathways is a critical question. Analysis of patients with psoriasis resistant to multiple Th17-pathway inhibitors (including anti–TNF, anti–IL-17s, anti–IL-12/23, and anti–IL-23 therapies) revealed that skin lesions expressed markedly elevated levels of IFN-α/β compared with those of responder patients (Saidoune et al 2026, in preparation). Quantitative analyses demonstrated that this type I IFN expression in resistant lesions was greater than the Th17 profile expression, indicating that the switch to dominance of the type I IFN pathway underlay the treatment resistance. Importantly, the enhanced type I IFN response was absolute and not a consequence of diminished Th17 activity. On the contrary, type I IFN dominance was accompanied by the concomitant upregulation of Th17 pathways along with Th1 and Th2 transcriptional programs, indicating that type I IFN drives broad compensatory immune activation (Saidoune et al, 2026 in preparation).

In AD, resistance to Th2-targeted therapies is similarly associated with a shift toward a non-Th2 pathway. Seremet et al, (2024) showed an increased Th1 response with a shift to dominance over Th2 pathways in patients with AD resistant to anti-IL4R blockade. Fukushima-Nomura et al (2025) demonstrated enhanced Th17 activation in patients with AD who fail anti-Th2 therapy. Whether the differences observed between these studies reflect ethnic variation in patient populations or distinct disease trajectories remains to be determined. More broadly, the molecular mechanisms driving immune reprogramming toward alternative dominant pathways and that confer therapeutic resistance remain incompletely understood.

## Immune Shifts and Phenotype Switches in Paradoxical Reactions

Beyond therapeutic resistance, immune shifts are also observed in therapy-induced paradoxical reactions. These reactions can be defined as phenotype switches that emerge despite an initially effective therapeutic response, often manifesting at anatomical sites distinct from the original lesions (Maronese et al, 2024). The prototypical example is anti-TNF–induced paradoxical psoriasis, which occurs in approximately 5% of treated patients and typically presents with psoriasiform lesions involving the palms, soles, and scalp. These lesions are characterized by a pronounced type I IFN signature driven by the persistent activation of pDCs, which fails to resolve in the absence of TNF-mediated maturation signals (Conrad et al, 2018).

Other paradoxical reactions include phenotype switches from psoriasis to eczema during IL-17 blockade and from eczema to psoriasis during IL-4R blockade. Although commonly referred to as paradoxical reactions, these manifestations may rather represent the unmasking of an opposing disease phenotype under specific immune blockade conditions. Paradoxical eczema occurs in approximately 2–6% of patients with psoriasis and may reflect the unmasking of a latent atopic predisposition through an anti–IL-17A–induced compensatory immune shift from Th17- to Th2-driven inflammation (Al-Janabi et al, 2024; Chicharro et al, 2020; Michelucci et al, 2024). Conversely, IL-4 receptor blockade may induce a reciprocal shift from Th2- to Th17-mediated immunity, leading to psoriasiform plaques in patients with AD (Ali et al, 2022; Casale et al, 2022; Napolitano et al, 2019; Su and Zeng 2023; Trave et al, 2023). More recently, bullous pemphigoid–like paradoxical reactions have been reported in patients receiving IL-23 and IL-12/23 blockade (Guern et al, 2015; Xi et al, 2025; Zhang et al, 2023). This paradoxical reaction may represent true pemphigoid disease with autoantibody formation against BP180 and BP230, linear Ig deposition along the dermoepidermal junction, and subepidermal blistering. Transcriptomic analyses suggest a shift from Th17 dominance toward Th2 dominance with an additional codominant macrophage signature (Cometto et al, submitted), consistent with the molecular profile of classical bullous pemphigoid (Seremet et al, 2024).

## The Concept of Immune Continuum and Immune Shifts

Collectively, these observations support the concept of an immune continuum governed by a dynamic equilibrium between major inflammatory pathways. Under physiological conditions, Th17, Th2, Th1, type I IFN, and neutrophilic immune programs coexist in a tightly regulated balance, with reciprocal inhibitory and reinforcing interactions that maintain cutaneous immune homeostasis.

Figure 3 illustrates the balance between these immune programs. The type I IFN pathway acts as an upstream initiator of the Th17 program in psoriasis by promoting myeloid dendritic cell activation with the production of TNF and IL-23, leading to subsequent Th17 polarization. Within the Th17 pathway, TNF suppresses type I IFN expression (Palucka et al, 2005), whereas IL-17A and IL-17F inhibit keratinocyte-derived TSLP, a key epithelial cytokine that drives Th2 immunity (Bogiatzi et al, 2012). Conversely, within the Th2 program, IL-4 and IL-13 inhibit dendritic cell production of IL-23 and IL-12 (Guenova et al, 2015), thereby restraining Th17- and Th1-mediated inflammation, respectively.

**Figure 3:**
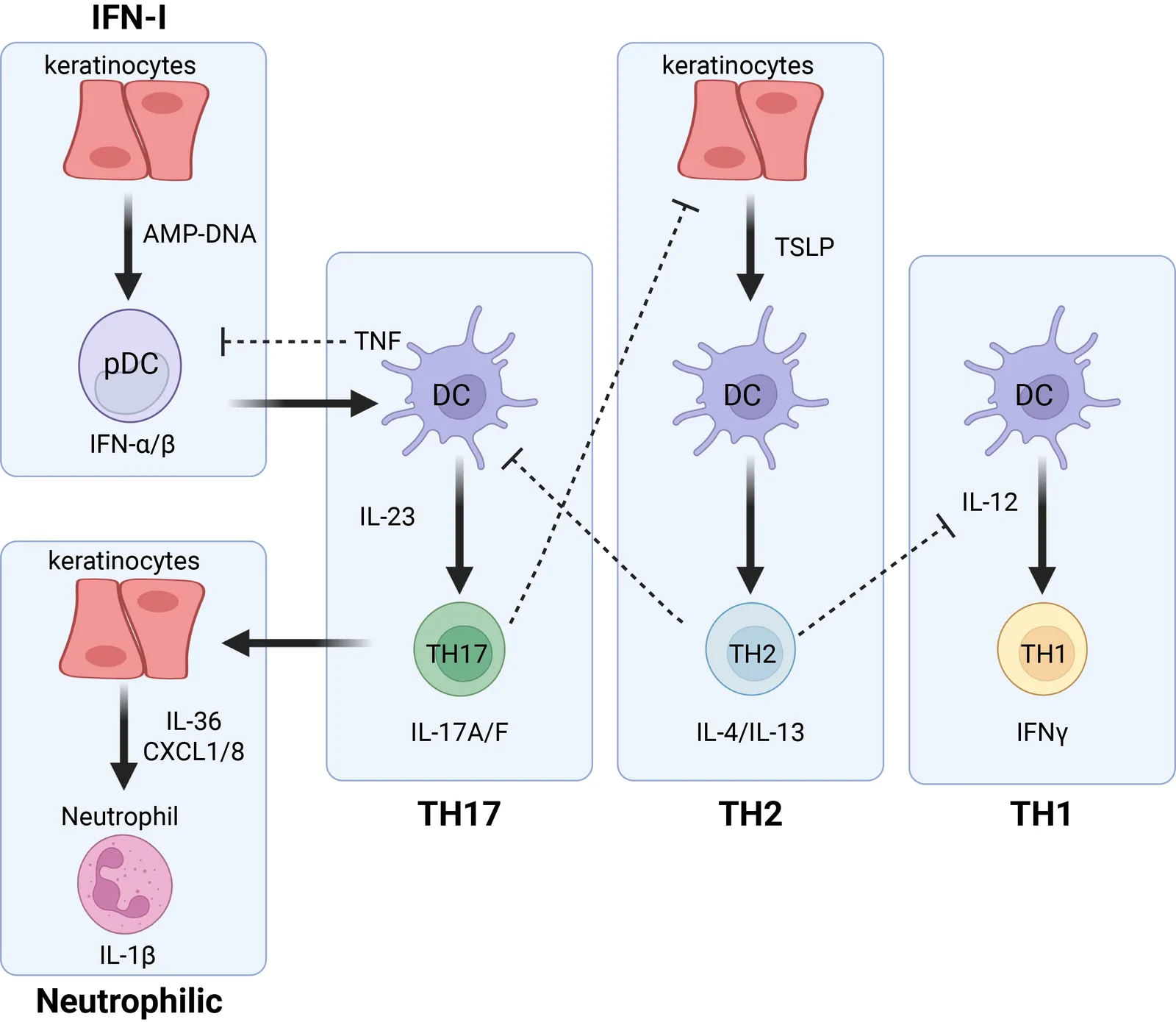
**Integrated regulation of type I IFN, neutrophilic, Th1, Th2, and Th17 immune responses during skin inflammation.** The immune pathways described in Figures 1 and 2 are depicted here as interconnected modules to illustrate their dynamic balance and reciprocal regulation during skin inflammation. During a Th17 response, DC-derived TNF suppresses type I IFN production by pDCs, whereas IL-17 cytokines enhance epidermal barrier function. In contrast, during a Th2 response, IL-4 and IL-13 inhibit DC production of IL-23 and IL-12, thereby attenuating Th17 and Th1 responses, respectively. Innate and adaptive pathways are closely interconnected. Type I IFN signaling promotes Th17 immunity, whereas IL-17 cytokines support neutrophilic inflammation. Together, these cross-regulatory mechanisms shape the dominant immune responses in the skin. AMP, antimicrobial peptide; DC, dendritic cell; pDC, plasmacytoid dendritic cell; Th, T helper.

This delicate immune balance may shift under certain conditions (Figure 4). A spontaneous Th17-to-type I IFN shift has been observed in resistant psoriasis. Immune shifts may also occur under targeted therapies, in which sustained pathway-specific inhibition unveils latent inflammatory programs within the same individual. Therapy-induced immune shifts include Th17-to-type I IFN transitions under TNF blockade (Conrad et al, 2018), Th17-to-Th2 transitions under IL-17 blockade, and Th17-to-Th2 + macrophage transitions under IL-23 blockade. These immune shifts may lead to phenotype switches and the emergence of paradoxical reactions in predisposed individuals.

**Figure 4:**
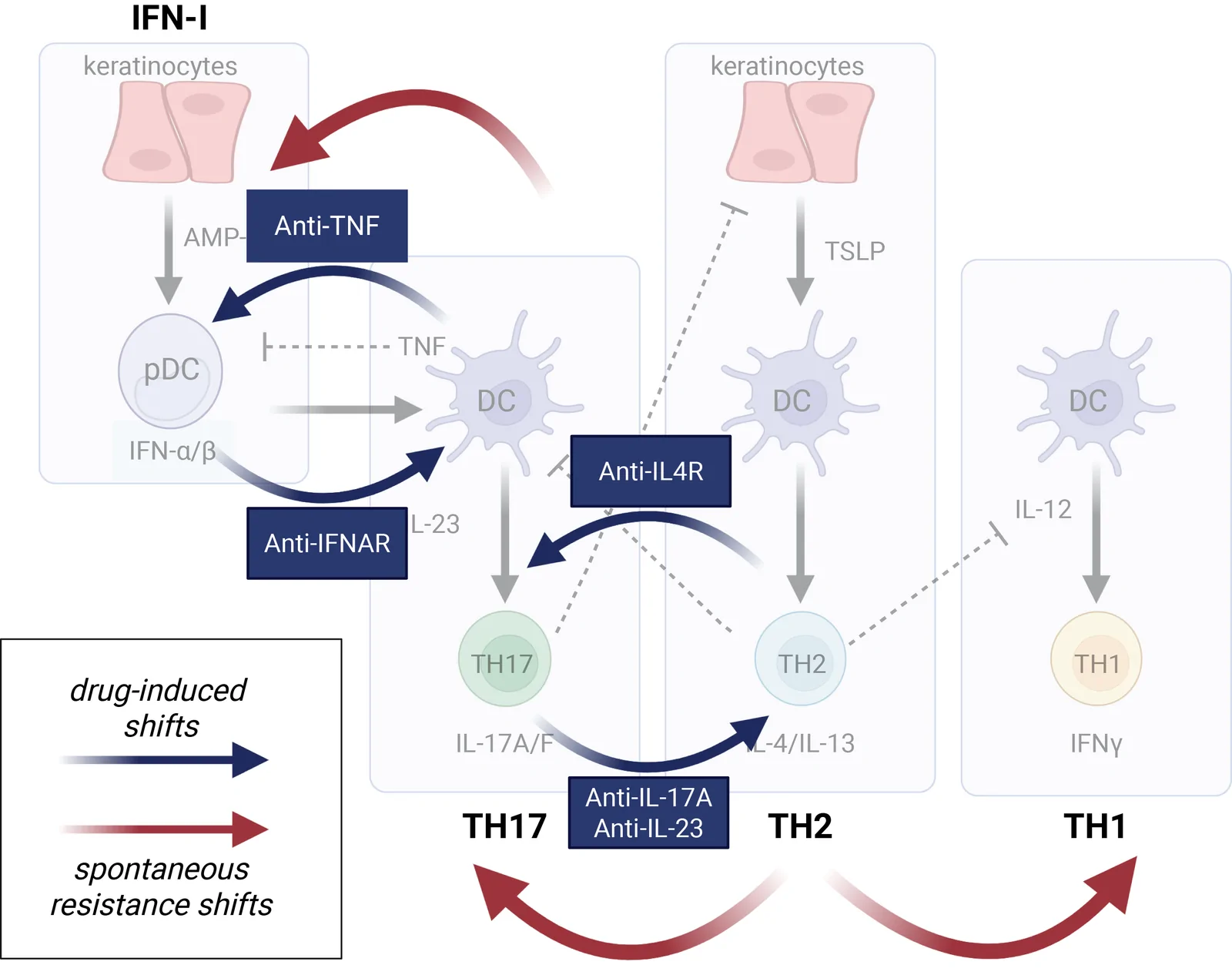
**Biologic targeting of inflammatory pathways may promote immune imbalance and paradoxical reactions.** Blockade of key mediators within dominant immune pathways can disrupt cross-regulatory mechanisms, resulting in shifts in immune responses. TNF inhibition maintains pDCs in a type I IFN–producing state. In contrast, IFNAR inhibition redirects the immune response from type I IFN production toward Th17-driven inflammation. Similarly, blockade of the Th2 pathway with anti–IL-4R antibodies enhances Th17 polarization. Conversely, inhibition of the Th17 axis through IL-23 or IL-17A blockade can impair epidermal barrier integrity and promote Th2-skewed responses. Comparable immune shifts are observed spontaneously in treatment-resistant patients: resistant psoriasis is associated with a Th17-to–type I IFN shift, whereas resistant eczema demonstrates a transition from Th2 toward Th1 or Th17 dominance. Blue arrows indicate biologic-induced immune shifts. Red arrows represent spontaneous immune shifts observed in treatment-resistant patients. DC, dendritic cell; pDC, plasmacytoid dendritic cell; Th, T helper.

In AD, a Th2-to-Th1 or Th2-to-Th17 transition has been reported in therapy-resistant disease. Furthermore, Th2-to-Th17 shifts leading to paradoxical psoriasis have been described under IL-4 receptor blockade. Interestingly, one report in lupus suggests a type I IFN-to-Th17 shift after IFNAR blockade, resulting in paradoxical psoriasiform reactions (Russo et al, 2025), suggesting a reciprocal control of these pathways.

## Therapeutic Implications

Immune shifts pose a major therapeutic challenge. Although highly selective biologics have transformed the management of inflammatory skin diseases, unidirectional pathway blockade may destabilize immune equilibrium, amplifying alternative inflammatory circuits. Furthermore, the use of additional single-pathway blockade to treat paradoxical reactions carries a substantial risk of reactivating the original disease, resulting in “flip-flop” phenomena (Müller et al, 2024). These phenomena are observed in patients with psoriasis who develop anti–IL-17–associated paradoxical eczema: treatment with Th2 blockade may re-exacerbate the underlying Th17-dominated psoriasis. Conversely, paradoxical Th17 reactions to anti-IL4R treatment should not be treated with anti–IL-17 because they are associated with a high risk of exacerbating the underlying AD.

Treatment of immune shift–driven disease may therefore require broader immunomodulatory strategies that simultaneously address both the primary disease and the newly dominant immune pathway. In this context, molecular immune profiling becomes essential to identify the operative inflammatory program at the time of treatment failure or paradoxical reaction and to guide appropriate therapeutic selection. In addition to conventional immunosuppressants, small-molecule inhibitors targeting multiple immune pathways, such as Jak inhibitors, which modulate Th1, Th2, and Th17 signaling, may be particularly effective in resistant (Seremet et al, 2024) and paradoxical (Fu et al, 2025; Salvi et al, 2024; Zhang and Jiang, 2024) disease states. However, Jak inhibitor–induced pustular psoriasis (Di Domizio et al, 2020; Shibata et al, 2019) illustrates that blocking broad adaptive T-cell responses can allow untargeted innate immune pathways to become dominant, leading to paradoxical neutrophil-driven inflammatory reactions.

TYK2 inhibitors may be preferable in type I IFN–dominant psoriasis, including both therapy-resistant disease and anti-TNF–induced paradoxical psoriasis, owing to their combined effects on type I IFN and IL-23/Th17 signaling. Clinical studies evaluating this approach in paradoxical psoriasis are currently ongoing (ClinicalTrials.gov identification NCT06281106; TYK2 Inhibition in Paradoxical Psoriasis, 2025). For resistant cases, the selection of such an approach should be informed by molecular confirmation of type I IFN pathway dominance rather than being inferred from clinical presentation alone.

Future therapeutic approaches may include multispecific antibodies or rational combinatorial strategies designed to re-establish immune equilibrium along the inflammatory continuum. The integration of molecular immune profiling into clinical decision making will be critical for implementing adaptive, precision-based therapies that restore immune balance and minimize therapeutic cycling.

## Summary and Perspectives

Inflammatory skin diseases are increasingly recognized as dynamic immune disorders that reside along a continuous molecular landscape rather than as fixed entities defined by a single dominant pathway. Accumulating evidence demonstrates that immune dominance can shift over time, either spontaneously or under targeted therapeutic pressure, leading to treatment resistance and phenotype switching. Although paradoxical reactions represent extreme manifestations of such immune shifts with overt phenotypic change, immune reprogramming frequently occurs without clear clinical correlates. This limitation underscores the shortcomings of phenotype-based disease management and positions molecular immune profiling as an essential tool to detect changes in immune pathway dominance and to guide appropriate therapeutic adaptations. Integration of immune profiling into routine clinical practice has the potential to enable adaptive, precision-based treatment strategies that restore immune equilibrium, minimize therapeutic cycling, and improve long-term disease control across the spectrum of inflammatory skin diseases.

## ORCIDs

Hugo Cometto: http://orcid.org/0009-0007-4267-5734

Fanny Saidoune: http://orcid.org/0009-0002-9776-6370

Jeremy Di Domizio: http://orcid.org/0000-0002-6281-3918

Michel Gilliet: http://orcid.org/0000-0002-4609-3762

## Conflict of Interest

JDD received a grant from Incyte and acted as a consultant for Signal26 Biotherapeutics. MG received grants from or acted as a speaker, consultant, advisory board member for AbbVie, Almirall, Amgen, AstraZeneca, Beiersdorf, Eli Lilly, Galderma, Johnson & Johnson, Kyowa Kirin, L'Oréal, Louis Widmer, Merz Pharma, Novartis, Permamed, Pfizer, Pierre Fabre, Sanofi, UCB Pharma, Ultrasun, Verfora, Wellzia, Biogen, Merck, AVVA, BMS, Galderma, Innate Pharma, Owkin, and Takeda. The remaining authors state no conflict of interest.
